# Double Trouble in the Thyroid: Coexistence of Hurthle Cell Neoplasm and Microfilarial Infestation

**DOI:** 10.15190/d.2025.14

**Published:** 2025-09-30

**Authors:** Neha Singh, Sujata Agrawal, Shrimayee Saha

**Affiliations:** ^1^Department of Pathology, Institute of Medical Sciences, Banaras Hindu University, Varanasi, Uttar Pradesh, India

**Keywords:** Microfilaria, Wuchereria bancrofti, Thyroid nodule, Fine-needle aspiration cytology.

## Abstract

Lymphatic filariasis, though often asymptomatic, remains a pervasive parasitic disease in endemic regions worldwide. The incidental identification of microfilariae on fine-needle aspiration cytology (FNAC) smears is rare and their coexistence with thyroid neoplasms is exceedingly uncommon. To our knowledge, very few cases of thyroid malignancy with coexistent microfilarial infestation have been documented. We describe a compelling case of a 68-year-old female who presented with a thyroid nodule and underwent FNAC. Cytological analysis revealed features diagnostic of a Hürthle cell neoplasm, accompanied by the unexpected presence of Wuchereria bancrofti microfilariae. Remarkably, the patient exhibited no clinical signs of filarial infection, and peripheral blood examination showed neither microfilaremia nor eosinophilia. This rare cytological finding underscores the diagnostic breadth of FNAC, not only in the evaluating thyroid pathology but also in revealing occult parasitic infestations. It highlights the necessity of meticulous smear evaluation, especially in patients from endemic regions presenting with chronic nodular lesions. The detection of neoplastic and parasitic elements together illustrates the complex interplay between infectious and neoplastic processes and reaffirms FNAC’s utility in identifying unexpected pathological associations.

## INTRODUCTION

Lymphatic filariasis is a vector-borne parasitic infection caused primarily by Wuchereria bancrofti and remains a significant cause of morbidity in endemic regions. While microfilariae are typically detected in peripheral blood, their incidental identification in cytological smears, particularly from non-lymphatic sites, is rare and often unexpected. Fine-needle aspiration cytology (FNAC) plays a crucial role in evaluating thyroid nodules, but the coexistence of parasitic infestation with thyroid neoplasms is exceedingly uncommon^1-5^. Here, we report a unique case of Hürthle cell neoplasm in which microfilariae were identified on FNAC in the absence of clinical or hematological evidence of filariasis.

## CASE PRESENTATION

**Figure 1:**
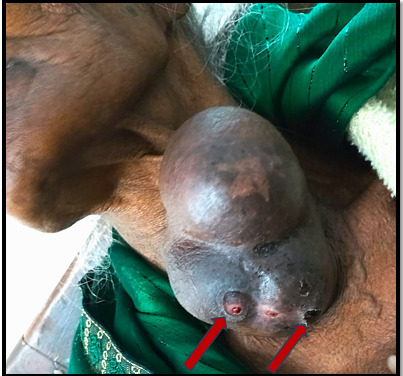
Clinical photograph of a 68-year-old female with a multilobulated anterior neck swelling consistent with thyroid enlargement. The mass extends across the midline and moves with deglutition but not with tongue protrusion, suggesting a thyroidal origin. The overlying skin is stretched and hyperpigmented, with an ulcerated area (marked with arrow) showing surface necrosis and oozing of blood-tinged colloid material. The lesion exhibits irregular nodularity with a firm-to-hard consistency, without clinically evident cervical lymphadenopathy or retrosternal extension.

A 68-year-old female presented with a progressively enlarging anterior neck swelling of one-year duration. The swelling was insidious in onset and gradually increased in size. There was no history of trauma, prior surgery, dysphagia, dyspnea, stridor, voice change, weight loss, night sweats, substance use or comorbidities. No systemic manifestations of filariasis, such as fever, lymphoedema, or elephantiasis, were noted, and there was no relevant family history. However, the patient resided in an endemic zone for microfilarial infection. On examination, a multilobulated, firm-to-hard, solid-cystic nodular mass measuring approximately 10 × 8 cm over the anterior neck. The swelling moved with deglutition but not with tongue protrusion. A prominent skin ulceration was noted, with active oozing of blood-mixed colloid from the surface (Figure 1).

Contrast-enhanced CT (CECT) of the neck revealed a large, ill-defined, heterogeneously enhancing solid-cystic mass measuring 6.8 × 7.6 × 8.4 cm involving the isthmus and left lobe of the thyroid. Areas of interspersed calcification were seen, along with disruption of fat planes with adjacent neck musculature and trachea. No significant lymphadenopathy or distant metastasis was identified. The lesion was categorized as likely neoplastic (TI-RADS 4).

**Figure 2:**
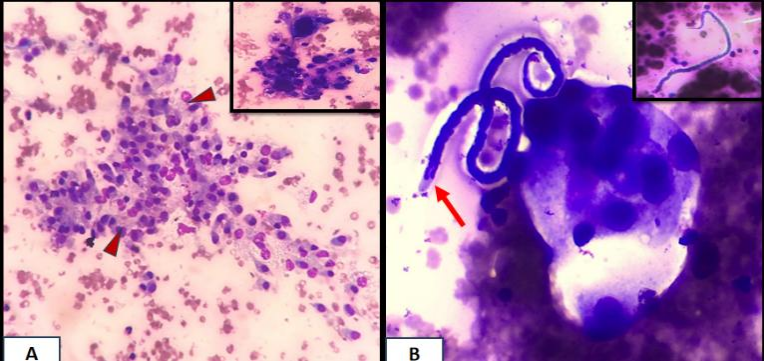
(A) Cytology smear from a thyroid swelling showing cohesive clusters of follicular cells with a high nuclear-to-cytoplasmic ratio, hyperchromatic nuclei, and oncocytic cytoplasm, admixed with macrophages (H&E, 40×, arrowhead). Inset: High-power view highlighting nuclear pleomorphism. (B) Smear showing a sheathed microfilaria of Wuchereria bancrofti clinging to thyroid follicular cells (Giemsa, 40×). The organism is slender, elongated, and thread-like, measuring approximately 240–300 μm in length and 7–10 μm in breadth. The sheath appears as a delicate, lightly stained covering around the parasite. The somatic nuclei are discrete, small, and uniformly arranged along the body, extending close to the tip of the tail but not reaching the extreme terminal end (arrow). Inset: Another field demonstrating a sheathed microfilaria in a colloid-rich background.

Fine-needle aspiration cytology (FNAC) was carried out using a 20-ml disposable syringe and a 23-gauge needle. The smear was dried and wet-fixed in 95% ethyl alcohol and stained by May-Grünwald-Giemsa and Papanicolaou stains, respectively. Cytology demonstrated moderate cellularity, with thyroid follicular epithelial cells in sheets, microfollicles, and clusters. The follicular cells were round to polygonal with abundant granular eosinophilic cytoplasm, well-defined cell borders, and centrally to eccentrically placed round nuclei with prominent nucleoli. The background showed colloid, hemorrhage, and cystic macrophages. Incidentally, a few sheathed microfilariae with gracefully curved bodies and pointed tails were identified in the background, morphologically consistent with Wuchereria bancrofti. A diagnosis of Hürthle cell neoplasm (Bethesda Category IV: Follicular neoplasm/Hürthle cell type) with an incidental finding of microfilariae was rendered (Figure 2).

Her complete blood count showed normal hemoglobin (12 g/dL;normal: 13–17 g/dL). Total leukocyte count was elevated(13.7×10³/μL;normal: 4–10×10³/μL), with absolute neutrophilia. Percentage and absolute eosinophil counts were normal: 4.73% and 0.65×10³/μL (normal: 1–6% and 0.2–1×10³/μL, respectively). Peripheral blood did not show any microfilariae. Thyroid function tests were also within normal limits.

The patient was advised to undergo thyroidectomy, but owing to advanced age and frailty, she declined surgical intervention. Medical management with diethylcarbamazine (DEC) was initiated. Unfortunately, the patient succumbed to her illness two months after the diagnosis, likely due to advanced age and disease-related complications.

## DISCUSSION

Filariasis, caused predominantly by Wuchereria bancrofti and Brugia malayi, continues to pose a major public health challenge in tropical and subtropical regions, with India bearing the largest disease burden worldwide^1,2^. While the lymphatic system is the classical site of involvement, microfilariae have occasionally been reported in aspirates from diverse anatomical locations including skin, subcutaneous tissue, breast, lymph nodes, bone marrow, effusion fluids, and rarely, the thyroid^3-5^. Their detection in fine-needle aspiration cytology (FNAC) is almost always incidental, particularly in patients lacking classical clinical features or peripheral microfilaremia.

The thyroid gland is an unusual site for microfilarial localization. In most reports, microfilariae have been identified in association with benign thyroid lesions such as colloid goitre or nodular hyperplasia^5-7^. Coexistence with neoplastic thyroid lesions, however, is exceptionally rare^4,8^. Pal et al. documented cases where microfilariae were observed in aspirates from malignant lesions across organs, including the thyroid, underscoring the possibility of chance associations^5^.

Several hypotheses have been proposed to explain the presence of microfilariae within neoplastic tissues:

- Altered tumor vasculature - Neoplasms are characterized by increased and aberrant vascularity. This irregular vascular network may entrap circulating microfilariae, facilitating their localization in tumor tissue.
- Immune microenvironment - Tumors often exhibit local immune dysregulation. Such an immunosuppressive milieu could permit the survival of microfilariae that might otherwise be cleared.
- Mechanical factors - FNAC procedures may induce minor vascular disruption, releasing microfilariae into the aspirate smear.
- Chance coexistence - Given the high prevalence of occult filariasis in endemic regions, incidental overlap with thyroid pathology remains a plausible explanation.

In our case, the absence of peripheral eosinophilia and lack of demonstrable microfilaremia favor either vascular entrapment or chance coexistence rather than systemic parasitaemia. This aligns with reports by Rekhi et al. and others, who described thyroid aspirates positive for microfilariae despite negative peripheral blood findings^6,9,10^.

Although histopathology often fails to reveal microfilariae in corresponding sections^4,11^, fine-needle aspiration cytology (FNAC) has repeatedly proven its utility in detecting these parasites at unsuspected sites. Meticulous examination of smears is, therefore, essential, particularly in endemic areas, to avoid overlooking such findings. In the present case, cytological detection ensured prompt recognition of concurrent filariasis, even in the absence of clinical suspicion or peripheral blood evidence. From a clinical perspective, such incidental detection carries therapeutic relevance. While the primary thyroid pathology generally dictates surgical management, the coexistence of filarial infection necessitates specific antiparasitic therapy, most commonly diethylcarbamazine (DEC), to eradicate occult infection and prevent progression^2,12^. Failure to identify this dual pathology may result in missed opportunities for appropriate treatment, underscoring the indispensable role of FNAC in identifying both neoplastic and parasitic pathology and ensuring comprehensive patient care.

## CONCLUSION

This case is significant for several reasons. It documents the rare occurrence of microfilariae within a thyroid neoplasm, highlights that such detection is possible even in the absence of peripheral blood or clinical evidence of filariasis and emphasizes the role of FNAC in simultaneously identifying neoplastic and parasitic pathology. For clinicians and pathologists working in endemic regions, the case reinforces the importance of meticulous cytological evaluation of all aspirates, as it may reveal dual pathologies with implications for both diagnosis and therapy. Ultimately, while the coexistence of microfilariae with thyroid tumors is most likely incidental, its recognition is essential for comprehensive patient care and adds valuable insight to the spectrum of thyroid cytology.
